# Supplementary material for: A van der Waals antiferromagnetic topological insulator with weak interlayer magnetic coupling
Source: Nat Commun. 2020 Jan 7;11:97. doi: 10.1038/s41467-019-13814-x (PMC6946652; doi:10.1038/s41467-019-13814-x)
Supplement: Supplementary file 1 — Supplementary Information [file 41467_2019_13814_MOESM1_ESM.pdf]

Supplementary Information: A van der Waals antiferromagnetic topological insulator with weak interlayer magnetic coupling

Hu et al.

Supplementary Note 1: Structure determination and transport measurements

Supplementary Figure 1. Powder X-ray Diffraction of $MnBi_4Te_7$.

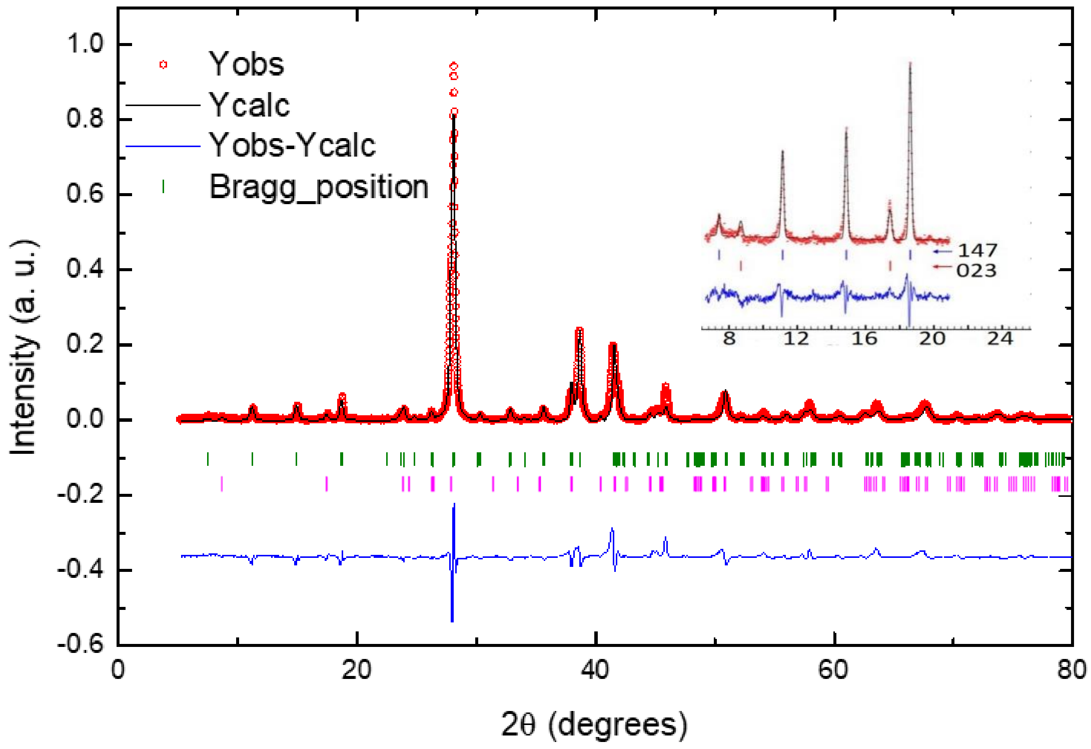

(a) Powder XRD spectrum and the refinement of $MnBi_4Te_7$. 147: $MnBi_4Te_7$, 023: embedded Bi_2Te_3 flux inside the crystal. Inset: the zoom-in plot of the powder X-ray below 22 degrees.

Single crystals grown using flux method have flux embedded inside. To make a quantitative estimation of the amount of Bi_2Te_3 flux embedded inside the single crystals, high quality powder X-ray diffraction pattern is needed. Several large single crystals were cleaved to remove the top- and bottom- surface Bi_2Te_3 flux. These pieces were then ground into powder where powder X-ray diffraction was made. The powder XRD is shown in Supplementary Figure 1. The Rietveld refinement suggests that in a $MnBi_4Te_7$ single crystal, Bi_2Te_3 is the only impurity phase. Because $MnBi_4Te_7$ and Bi_2Te_3 have similar reflections for non-(00l) peaks, so only (00l) peaks are good for the refinement to determine the relative molar ratio between them. Refinement in this region shows the embedded Bi_2Te_3 flux is around 14% inside single crystals. This

percentage is used to correct the value obtained in our magnetic property measurements. Supplementary Table 1 and Supplementary Table 2 summarize the detailed crystal structure of $MnBi_4Te_7$ obtained from the refinement of our powder X-ray diffraction pattern.

Supplementary Table 1. Rietveld refinement results of powder $MnBi_4Te_7$ at room temperature.

**Results**	**$MnBi_4Te_7$**
Space group	P-3m1
a (Å)	4.3454(5)
c (Å)	23.706(4)
V (Å^3)	387.65(9)
Reliability factor Rp	28.8

Supplementary Table 2. Refined structural parameters for $MnBi_4Te_7$ at room temperature based on the powder x-ray diffraction data. The B factor was fixed at 0.8 for all atoms.

**Atom**	**Site**	**x**	**y**	**z**	**B**
Mn	1b	0	0	0.5	0.8
Bi1	2d	0.3333	0.6667	0.0866(4)	0.8
Bi2	2d	0.3333	0.6667	0.3407(4)	0.8
Te1	1a	0	0	0	0.8
Te2	2d	0.6667	0.3333	0.1546(7)	0.8
Te3	2c	0	0	0.2671(7)	0.8
Te4	2d	0.6667	0.3333	0.4355(6)	0.8

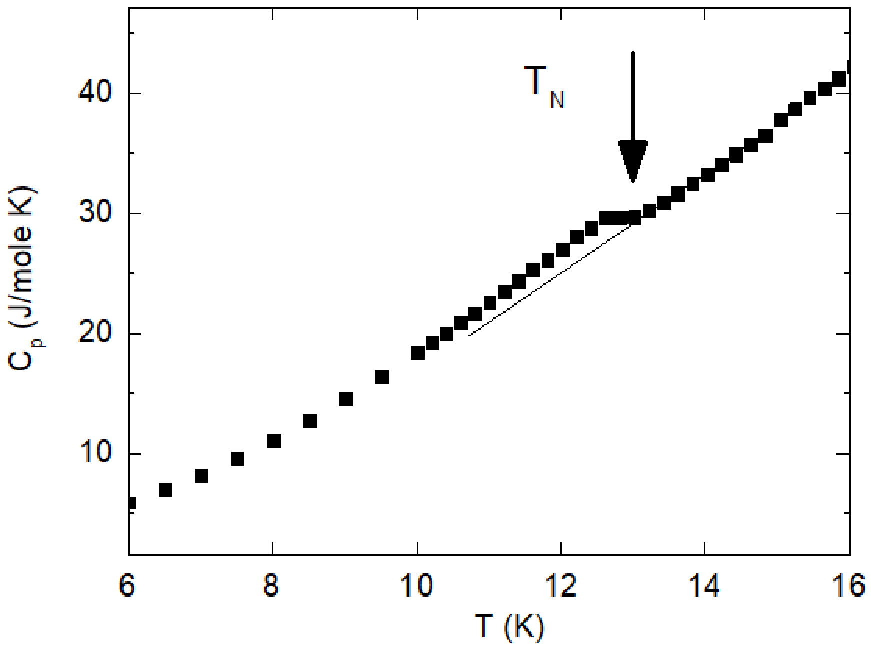

Supplementary Figure 2: Specific Heat of $MnBi_4Te_7$ at zero field.

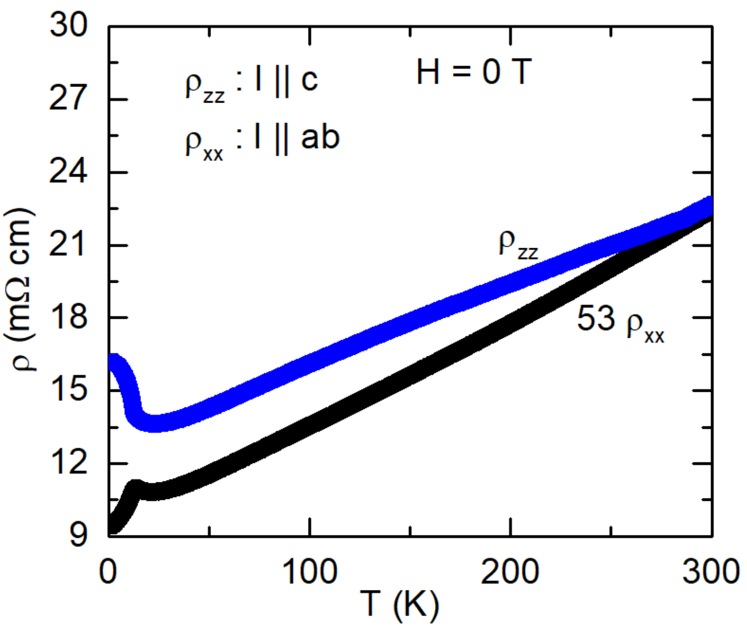

Supplementary Figure 3: Temperature dependent resistivity $\rho_{xx}(T)$ (I // ab) and $\rho_{zz}(T)$ (I // *c*) at zero field.

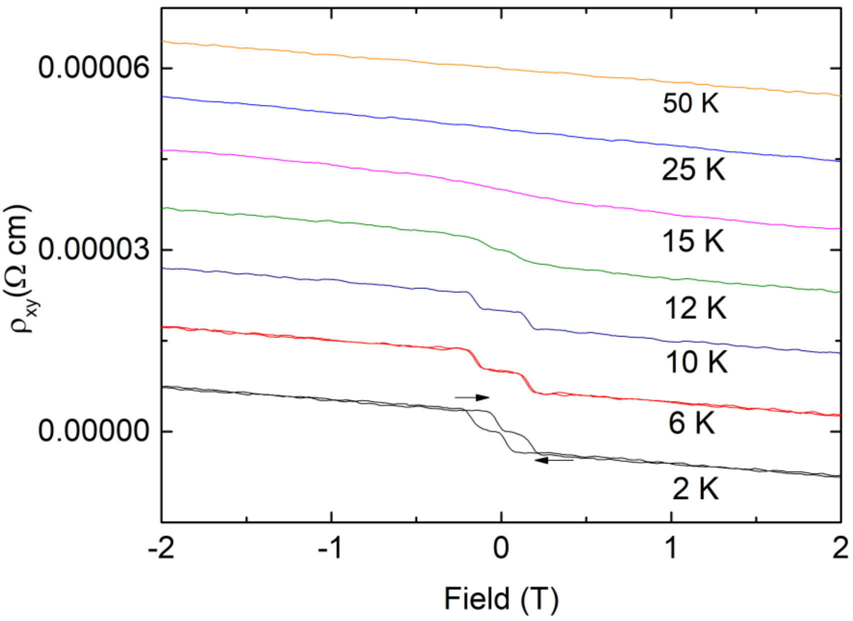

Supplementary Figure 4. Bulk Hall resistivity $\rho_{xy}(H)$ with I // *ab* and H // *c* under different temperatures. All curves are centered at 0, but shifted relatively for better comparison.

Supplementary Figure 2 shows Heat Capacity measured at zero field near the Neel Temperature. Supplementary Figure 3 shows the temperature-dependent resistivity of $\rho_{xx}(T)$ (I // ab) and $\rho_{zz}(T)$ (I // *c*) measured at zero field from 2 to 300 K. Supplementary Figure 4 shows the bulk Hall Resistivity $\rho_{xy}(H)$. The hysteresis feature shown at 2 K vanishes near 6 K but the step-feature due to the spin-flip transition persists up to T_N. As temperature further increases above T_N, the step feature is broadened gradually with weaker spin fluctuations until ρ_{xy} becomes completely linear above 50 K.

Supplementary Note 2: Exchange coupling of bulk $MnBi_4Te_7$

$MnBi_4Te_7$ consists of one $MnBi_2Te_4$ layer and one Bi_2Te_3 layer alternately stacked. The magnetic moments of the system are on the Mn atoms. The exchange coupling constants J for $MnBi_4Te_7$ are derived from the Ising model $H = -\sum_{(i,j)} J_{(i,j)} \sigma_i \sigma_j$,

where $\sigma_i \in \{1, -1\}$ is spin-up or spin-down at a specific site. For convenience, we use a $1 \times 1 \times 2$ cell to calculate the interlayer exchange coupling constants $J_\perp$ as shown in Fig. 1a in the main text, while use a $4 \times 3 \times 1$ cell to calculate the intralayer exchange coupling constants up to the fourth nearest neighbor as shown in Supplementary Figure 5. Our results are summarized in Supplementary Table 3.

The interlayer exchange coupling $J_\perp$ = -0.150 meV/Mn suggests that the magnetic configuration favors AFM stacking along the c axis. In addition, compared with $MnBi_2Te_4$, the value of $J_\perp$ is about one order of magnitude smaller than -1.4 meV/Mn, the counterpart of $MnBi_2Te_4$[2], suggesting that a much smaller magnetic field is required to stabilize the FM phase of bulk $MnBi_4Te_7$ or its 2D films. On the other hand, among the intralayer interactions J_1-J_4 the one between first nearest neighbors within the Mn sublattice is predominant (orders of magnitude larger than the other coupling constants), which indicates a FM in-plane magnetic configuration. The values of intralayer coupling constants are in good agreement with those reported by Ref[3].

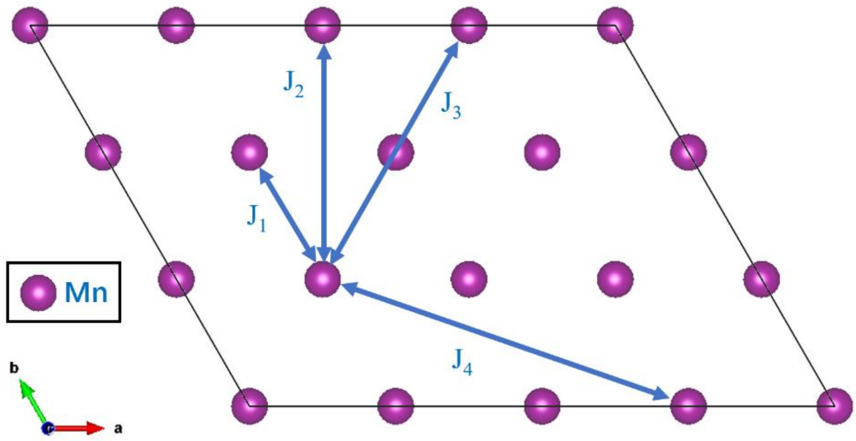

Supplementary Figure 5: The intralayer exchange coupling constants J_1, J_2, J_3 and J_4 connecting the first, second, third, fourth nearest neighbors in the Mn sublattice.

Supplementary Table 3: The exchange coupling constants J (meV/Mn) of $MnBi_4Te_7$. $J_\perp$ is the interlayer exchange coupling constants, while J_1, J_2, J_3 and J_4 are the intralayer exchange coupling constants connecting the first, second, third, fourth nearest neighbors, respectively.

J (meV/Mn)	$MnBi_4Te_7$
$J_\perp$	-0.150
J_1	1.704
J_2	-0.075
J_3	0.039
J_4	0.008

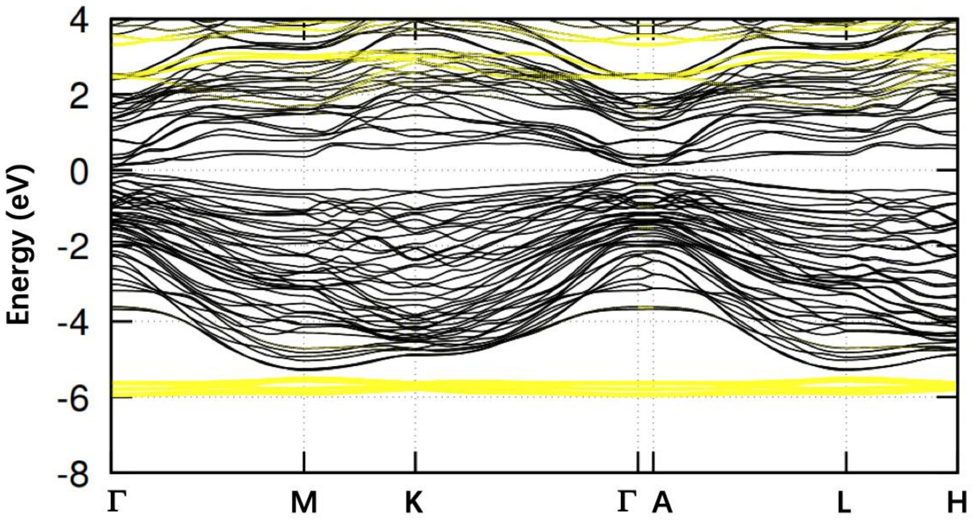

Supplementary Figure 6: Calculated band structure of bulk AFM $MnBi_4Te_7$ with the projection of Bloch eigenstates onto Mn-3*d* (yellow) orbitals.

Supplementary Figure 6 shows the DFT calculated band structure of bulk $MnBi_4Te_7$ with the A-type AFM. The projection of Bloch eigenstates onto Mn-3*d* orbitals is shown in yellow. It can be seen that Mn-3*d* states form nearly flat bands far away from the Fermi level.

Supplementary Note 3: ARPES measurements

Due to finite instrument resolution and the higher density of states above/below the Dirac point, it is possible for an intrinsically gapless system to show an “apparent” gap

in ARPES (Supplementary Figure 7). Therefore, we studied the surface state of the apparent Dirac cone on the [$MnBi_2Te_4$] SL termination carefully with numerical simulation. In our simulations, we estimate the Fermi velocity of the simulated Dirac cone from our data, and all instrument resolution functions were assumed to be Gaussian.

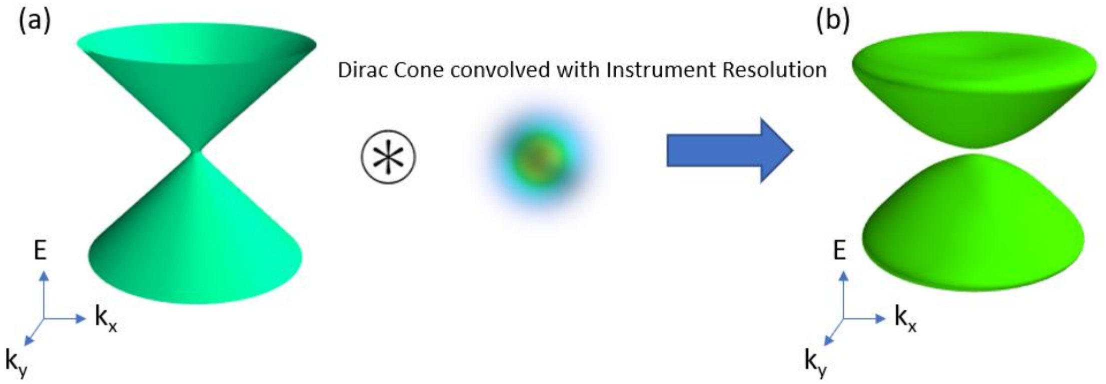

Supplementary Figure 7: An example of a typical isosurface generated in our simulation: (a) with only intrinsic self-energy Σ and (b) with experimental instrument resolution parameters.

We can simulate an experimental ARPES spectra due to a single, possibly gapped Dirac cone, using the ARPES spectral function,

$$A(E,k) = \frac{\mathrm{Im}(\Sigma)}{(E - E_0(k) - \mathrm{Re}(\Sigma))^2 + (\mathrm{Im}(\Sigma))^2}$$

where Σ is the complex self-energy of the electron due to the many body interaction and $E_0(k)$ is the bare band dispersion of a possibly gapped Dirac cone, $E_0(k) = \pm(\hbar v_{\mathrm{F}}\sqrt{k_x^2 + k_y^2} + \Delta/2)$, where Δ is the size of the gap. This spectral function is further modified by including the instrument resolutions, which are Gaussian functions convolved with the above spectral function. The energy resolution of the instrument σ_{E} is obtained by fitting the sample's Fermi edge to a Fermi function. The angular resolution of the instrument, σ_{angle}, is estimated from the sharpest momentum distribution curve (MDC) obtained, which was at the Dirac node, and we estimate $\sigma_{\mathrm{angle}} = 0.13°$. Although the coupling between Σ σ_{angle} can result in

some uncertainty in σ_{angle} in the estimation, σ_{angle} should not be smaller than the instrument manufacturer's specified angular resolution: $0.1°$.

During the simulation, we fix σ_{E} since σ_{E} arises from the presence of the sample's Fermi edge; we make σ_{angle}, Σ and Δ as the free parameters since Σ and Δ are unknown and σ_{angle} is coupled to Σ. Then we closely monitor σ_{angle} so that it is no smaller than the instrument manufacturer's specification. When we simulate a spectrum, there are several features we need to keep track of to ensure the simulation resembles the measured spectra: 1) the MDC linewidths, 2) the size of the measured gap, and 3) if there is a gap, the contrast of the gapped region to the peak of the bands above and below.

Our simulations help reveal the complex effect of each individual term has on the measured ARPES spectra. Increasing Σ broadens the APRES spectra primarily in energy. If the system is intrinsically gapped, a large Σ can reduce the contrast of the gap, making the gap appear smaller and even eliminating it if the gap is small enough. On the other hand, σ_{angle} is an important parameter that typically exaggerate the size of gap, or manifest one even if it does not exist.

Supplementary Figure 8(a-c) show examples of simulated ARPES *E-k* map, to be compared with 8(d) taken on the [$MnBi_2Te_4$] SL surface. We found that if the intrinsic gap Δ is greater than 10 meV, to produce a spectrum similar to our experimental data, σ_{angle} had to be set less than $0.1°$, the instrument manufacturer's specified angular resolution, which is unlikely. Furthermore, the gapped region begins to show significant contrast that we don't see in our ARPES experiment. Therefore, we conclude that the surface state on the [$MnBi_2Te_4$] SL has a gap smaller than 10 meV, if the gap exists at all.

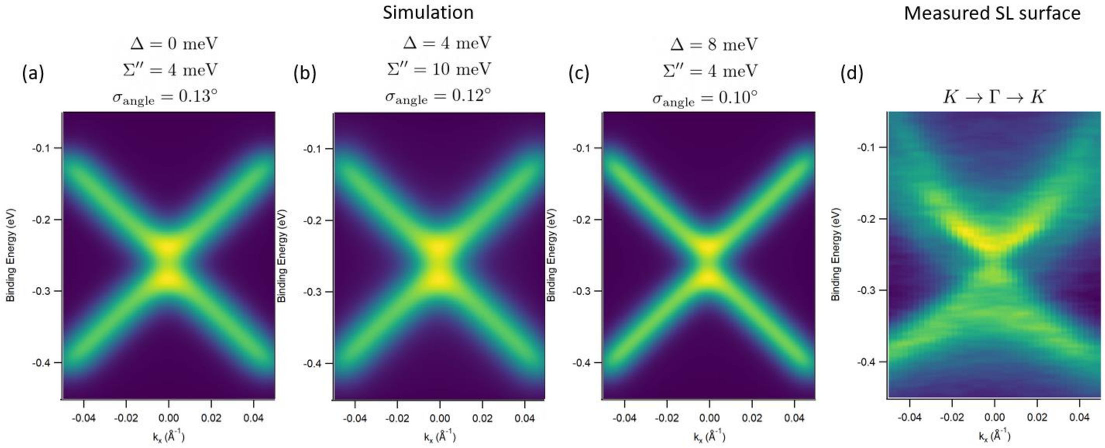

Supplementary Figure 8: (a-c) Simulated ARPES spectra of a single Dirac cone with Fermi velocity $\hbar v_F = 2.9\ eV$ Å and various other simulation parameters labelled above the plot, all with a measured peak-to-peak gap of approximately 44 meV. (d) Experimental ARPES on the [$MnBi_2Te_4$] SL surface in the vicinity of the Dirac cone.

References

[1] Aliev, Z. S. *et al.* Novel ternary layered manganese bismuth tellurides of the MnTe-Bi_2Te_3 system: Synthesis and crystal structure. *J. Alloys Compd.* (2019).

[2] Otrokov, M. *et al.* Unique Thickness-Dependent Properties of the van der Waals Interlayer Antiferromagnet $MnBi_2Te_4$ Films. *Phys. Rev. Lett.* **122**, 107202 (2019).

[3] Otrokov, M. M. *et al.* Prediction and observation of the first antiferromagnetic topological insulator. Preprint at https://arxiv.org/abs/1809.07389 (2018).
